# Criticism or Cash?

**Published:** 1889-02-16

**Authors:** 


### The Hospital

A Weekly Institutional Journal of

Science, Medicine, Nursing, and Philanthropy.

*For Hospitals, Asylums, Medical Practitioners, Students, Nurses, Families, Parishes Congregations, and the Charitable Public.*

Vol. V. No. 125.] [February 16, 1889.

### Criticism or Cash?

Both, by all means! Is there any pleasure in ill-done work, even to the doer of it? If the work be costly as well as ill done is not that a reason for dissatisfaction and prompt reformation? The critic is often feared, but seldom loved. That is not entirely the fault of the criticised. If the critic were absolutely impartial, generally wise, and always kindly he might come in time to be loved as well as feared. "Might" come to be loved we say; for it is not certain that he would. There are among human creatures a certain number who are so hopelessly and wilfully stupid and self-pleasing that nothing will content them but the fullest liberty to do exactly as they choose on all possible occasions. To these the wisest counsellor, the most impartial judge, the kindliest suggestor of different methods is always an exciter of angry passions and heated speech.

But is it or is it not true that lookers-on always see most of the game? Let a man picture to himself an actual game, say of cricket, in full progress. Mr. W. G. Grace might be a player or a spectator. If he were asked to give a full and critical report of any particular match, who can doubt whether he would prefer to be a player or a looker on? As a player he could not give a full, true, and particular account; as a spectator he could do it easily. Criticism is a part of teaching. The most distinguished natural geniuses, and the most painstaking workers, may alike be instructed and improved by it. And these are, as a rule, the very persons who welcome criticism most, provided, of course, that the critic be competent by education, experience, and judicial temper, to sit in judgment on their particular work. Mere fault-finding by incompetent persons cannot be called criticism. It is impertinence, and must be treated as such.

Great work requires for its well-doing great minds. All human work is the better the more mind there is put into it. But great work cannot be done at all except by great minds. Great place does not create great minds. A "Bumble" may be consecrated King or Archbishop, but he will still be a "Bumble." In a vast and widely influential Empire like ours there are many kinds of public work which may truly be called "great." The value of work must be measured by its intrinsic importance, by the number of individuals included within its range, by the relative amount of money which is shut up in it as capital, or which is annually circulated by it as revenue, and by a hundred other like considerations. Tried by these standards there are for the men and women of this Empire many kinds of public work of the first magnitude. The statesman fills the public eye at the moment more than any other individual, but his particular work at the time may be neither so great in itself nor so enduring in its consequences as that of the public teacher or of the philanthropist. What we all require to have made clear to us is this: that the very work which Providence may have assigned to us at any given time is worthy of our best powers of mind and conscience; and that it is only by the exercise of these best powers that any work at all can be done as it ought to be done.

If money constitutes worth, what must be the capital value of the hospitals of Great Britain, whose annual income is not less than five or six millions—a sum exceeding all the revenues of the State at so comparatively recent a period as the time of Queen Elizabeth and the Armada? If numbers of individuals confer importance, what must be the magnitude of the interests of hospitals when in London alone more than a million persons annually seek their aid? If work is to be measured by its intrinsic value, what other work, except indeed that of the production of food, without which man cannot live at all, can compare with the work of healing the sick and restoring the injured and dying to life and health? The general policy, the direction, the administration, the support, and the efficiency of such a comprehensive and costly organisation as the hospital system of this country is a work which is hardly second in the magnitude of its interests to any of the most important departments of the State. That being so, those who know, those who have had experience, must be critical. They would be false to the worthiest traditions of English life, nay, to the most elementary duties of manhood if they did not speak, and speak boldly, and freely all that is in their minds. That they will meet with opposition is certain; that they may be misjudged, charged with unworthy motives, denounced as enemies to the good cause—that also is inevitable. But what then? Will a man of truly noble and courageous mind permit the great work of public philanthropy, one of the noblest monuments of English character which the world has ever seen—will he permit it to be defaced, injured, and ruined, lest he should incur the anger and the vituperative speeches of persons whose minds never rise above a daily dinner, a comfortable bed, and a sufficient salary paid punctually on quarter day? If British public men are to come to such a craven temper as that, then indeed the days of all great causes are numbered, and work good in itself must be left in unworthy hands until it ceases to be good and is fit only to be destroyed.

What has raised hospitals to their present pre-eminence among the noble institutions erected by British philanthropy? Good work! What has made the word "hospital" synonymous with all that is highest in medical skill, all that is gentlest in nursing care, all that is most restful and hopeful as a refuge for the sick, the sorrowing, and the destitute? Good work! And is that noble edifice of benevolence and good deeds, reared with so much pains and self-denial, to be permitted to be undermined by careless management, and assaulted by self-seeking enemies in the guise of friends until it is brought into general contempt and ruin? The mere thought is unendurable. Not only must criticism be welcomed, but every hospital manager, every physician, every secretary, every matron, and even every nurse, should become the severe but friendly critics of their own institution, and resolve that in every detail of its domestic management and its professional work it shall be second to none in the world.

"Give us cash," say some, "and not criticism." Unreasoning wolves these, who want to eat whether they think and work or not. Criticism is the parent of cash, just as ploughing and sowing are the parents of harvest, and the week's work of the Saturday's wages. It is the experience of every hospital that good work, well and economically done, commends itself to the critical mind of the hospital supporter, and loosens his purse-strings more effectually than the most powerful appeal of the pulpit or the most eloquent effort of the press. The last quinquennial petition of the London Hospital was successful almost beyond anticipation. What was the reason? The managers relied on one argument, and on one only. "Here is our work," said they, "and this is the cost of it." The circular they issued was the briefest of the brief; it contained a record of facts. Lord Beaconsfield once said that "knowledge was the parent of eloquence." It is equally true that facts are the parents of conviction. Convince the English people, by incontestable facts, that good work is being done, and done at a moderate cost, and they will do everything that may reasonably be required for the support of such work. The best managed hospitals are not in straits. If ever they do fall into limited resources they do not need to fill the air, like Eastern beggars, with their distressful howlings. They make known their facts, and that is found to be amply sufficient. The best friends that hospitals have are those who bring to bear upon their management a critical mind and a kindly spirit. These are the persons to be encouraged by officials, and they are encouraged by all officials who have intelligence and honesty of purpose. These are they who are trusted by the public, because they are known to be both experienced and incorruptible. Such persons have our fullest sympathy. If they take the trouble to know and to criticise, they also exercise the self-denial which giving requires. The articles of their faith are two, not one. "Criticism" alone they do not believe in, nor do they hold with "cash" alone. Instead of separating they unite the two, and stand with unwavering confidence by the incontrovertible formula, "criticism *and* cash."

